# Effect of bilateral internal thoracic artery harvesting on deep sternal wound infection in diabetic patients: Review of literature

**DOI:** 10.1016/j.amsu.2021.102382

**Published:** 2021-05-07

**Authors:** Matiullah Masroor, Xianming Fu, Umar Zeb Khan, Yuan Zhao

**Affiliations:** a Department of Cardiovascular Surgery, The Second Xiangya Hospital of Central South University, 139 Renmin Middle Rd, Changsha, 410011, China; b Department of Cardiothoracic and Vascular Surgery, Amiri Medical Complex, Qargha Rd, Afshar, Kabul, Afghanistan; c Department of General Surgery, The Third Xiangya Hospital of Central South University, 138 Tongzipo Rd., Changsha, 410013, China

**Keywords:** Bilateral internal thoracic artery (BITA), Single internal thoracic artery (SITA), Deep sternal wound infection (DSWI), Diabetes mellitus (DM), Coronary artery bypass grafting (CABG)

## Abstract

Instead of its documented superiority of patency and long-term outcomes, the bilateral internal thoracic artery grafts are underused in the general population, and its use is controversial and debatable in diabetic patients due to long surgery duration, post-surgical bleeding, and sternal wound complications such as sternal wound infection, mediastinitis, and sternal wound dehiscence. This review article is particularly focused on deep sternal wound infection (DSWI) of bilateral internal thoracic artery (BITA) grafts in diabetic patients with comparison to single internal thoracic artery (SITA) graft.

## Highlights

• Bilateral internal thoracic artery grafting is superior to single internal thoracic artery grafting in terms of outcomes.
• Bilateral internal thoracic artery grafting is underused, especially in diabetic patients.
• The risk of DSWI in skeletonized BITA grafting in diabetic patients is almost similar to SITA grafting.
• Skeletonized BITA grafting should be encouraged in selected diabetic patients.

## 1 Introduction

Because of the widespread sedentary lifestyle throughout the world, especially in developed and developing countries, coronary artery disease is prevailing, which is requiring intervention at some point of life according to guidelines available. The available techniques for coronary revascularization are coronary artery bypass grafting (CABG), percutaneous coronary intervention (PCI) and, hybrid procedure (HCR) [1]. Arterial conduits for myocardial revascularization are getting popular during the last few decades because of their decisive advantages over classical venous conduits. Arterial conduits used for CABG surgery are internal thoracic arteries (ITAs), radial arteries, splenic artery, right gastroepiploic artery, ulnar arteries, and inferior epigastric arteries [2].

The use of BITA grafting during myocardial revascularization reportedly provides a survival benefit over SITA grafting [[3], [4], [5], [6], [7], [8], [9], [10]]. However, BITA grafting in diabetes mellitus patients is controversial because, it is believed to be a high risk for sternal infection.

40% of the patients undergoing CABG had diabetes, according to 2010 statistical data available [11]. The purpose of this review paper is to compare the DSWI rate of BITA grafting to that of SITA grafting in patients with diabetes.

## 2 Evidence about the superiority of arterial grafts over venous grafts

As the first attempt to revascularize the myocardium was the direct implantation of the left internal thoracic artery (LITA) into the anterior wall of the left ventricle by Arthur Vineberg in 1946 [12]. As we are at the end of the 5th decade of using BITA grafts in CABG surgeries for ischemic heart disease problems [13], arterial grafts have shown much better results than venous grafts, and that is why it has been prioritized over venous conduits [14]. According to the study, the use of BITA grafting is linked with decreased mortality, reoperation and percutaneous transluminal coronary angioplasty (PTCA) than SITA grafting [3]. Studies have confirmed that Nitric oxide (NO) derived from the endothelium of ITAs is more than NO derived from the saphenous vein. As NO helps in smooth muscle relaxation, avoid leukocyte sticking to the endothelium, disrupt activation of platelets and constrain the proliferation and migration of smooth muscles of the vessels. So, the contribution of increase NO to the patency and smooth flow of blood in blood vessels is evident [15]. During the early days of saphenous vein grafts in CABG surgeries, signs of its failure were becoming evident and pathological reports during the early 1970s showed thickening of the intimal and medial layer of saphenous vein and thrombosis of venous grafts. Other studies have shown that hyperplasia of the intima and premature atherosclerosis leads to a lower patency rate of venous grafts compare to arterial grafts [12].

## 3 Effect of internal thoracic arteries skeletonization on DSWI

Most researchers believe that the risk for DSWI will reduce with skeletonization, as skeletonization is a better way to preserve blood supply to the sternum. Deo et al. (2013), in a meta-analysis of 126,235 diabetic patients comparing DSWI in BITA vs SITA group, concluded that the risk of DSWI in diabetic patients undergoing CABG surgery could be minimized by skeletonized harvesting method, and much more attention should be paid to the preservation of sternal blood flow [16]. Rubens et al. (2015), in a retrospective cohort study of 1611 patients who underwent BITA graft surgery from January 2006 to December 2014, concluded that skeletonization plays an essential protective role in avoiding sternal wound complication. Although the skeletonized BITA patients were lower BMI in their study, they were significantly old age, diabetic, more proportion of women, renal failure, vascular disease, lung disease, low hemoglobin before surgery, and their surgeries were emergency compared to non-skeletonized BITA grafts surgery. With all given comorbidities skeletonized group had lower sternal complication rate, and it was concluded that skeletonization has protective role in SWI; still, there was no prominent effect of skeletonization in women [17]. A recent four-year study from January 2014 to December 2017 by Ji et al. (2020), on skeletonized BITA vs skeletonized SITA concluded that skeletonized BITA grafts have a similar risk of DSWI as skeletonized SITA graft. They further described the effect of skeletonization in subgroup diabetic and non-diabetic, which also showed similar risk for DSWI [18]. On the contrary, Lazar et al. (2018), in a review article covering the literature from 1970 to 2017, discussed the issue and concluded that DSWI is a multifactorial issue and skeletonization of ITAs have no effect over DSWI [19].

There is a clear controversy in these studies, but from our point of view and the evidence from other studies, skeletonization may play a role in avoiding SWI as a single factor. Factors such as female gender, old age, obesity, diabetes mellitus, chronic obstructive pulmonary disease (COPD), renal failure, peripheral vascular disease (PVD), and many more may also contribute to the development of sternal wound infection; suggesting that even in skeletonized ITAs if the aforementioned factors are not kept in consideration, there is a possibility of SWI development. Kieser et al. (2014) studied 1001 patients, of which 34% were diabetic, and their last 460 patients developed 0% DSWI. The measures they applied were ITA skeletonization, irrigation of wound, no bone wax, ITAs harvesting with a harmonic scalpel, one observer per case, sternal marrow vancomycin paste application, iodine-soaked skin drapes, skin preparation with chlorhexidine-alcohol, avoidance of BITA in obese diabetic women, aseptic wound care, more off-pump surgeries, and irrigation of sternal marrow before sternal bone approximation. According to their analysis, the irrigation of sternal marrow before sternal bone approximation less likely contributed to the prevention of DSWI. Chlorhexidine alcohol skin preparation and avoidance of BITA in obese diabetic women, which had 10-fold more risk for DSWI, were the key measures in preventing DSWI. Other diabetic patients, including obese diabetic men, had no increased risk of deep sternal wound infection [20].

## 4 Comparison of BITA vs SITA grafting from DSWI perspective in diabetic patients

Pevni et al. (2017) suggested in a large cohort study that the patients with diabetes and multi-vessel disease undergoing BITA grafting for ischemic heart disease have better long term outcomes than those diabetic patients undergoing SITA and saphenous vein graft for ischemic heart disease [4].

There is much evidence available in the literature that the long-term outcome of BITA grafts is better than that of pure venous grafts and even better than SITA combine with saphenous vein grafts in the general population as well as in diabetic patients [[4], [5], [6], [7], [8], [9], [10]]. The benefits of BITA grafting are also evident in high-risk patients compare to SITA grafting, such as patients with low EF, female, recent myocardial infarction, emergency surgeries, obese, old age, end-stage renal disease (ESRD), on hemodialysis, with PVD, and patients with COPD [[21], [22], [23], [24], [25], [26], [27], [28], [29], [30], [31], [32], [33]].

Despite all these benefits, surgeons seem to be much reluctant to perform BITA grafting in patients with multi-vessel CAD, especially in those with diabetes mellitus. According to statistical data available, less than 5% of patients undergo BITA in America, 12–20% in Europe, and 30% in Japan [20,34,35]. This procedure's reluctance is considered to be prolonged surgery time, post-surgical bleeding, and sternal complication (deep and superficial sternal wound infection, mediastinitis, and sternal wound dehiscence).

To the best of our knowledge, all the papers available in the English language on PubMed and google scholar comparing BITA vs SITA and its effect on DSWI in diabetic patients are mentioned in Table 1 [4,[6], [7], [8], [9],^18^,[36], [37], [38], [39], [40], [41], [42], [43], [44], [45], [46], [47], [48], [49], [50]]. Most of the studies cited in Table 1 supported the idea of BITA grafting in diabetic multi-vessel CAD patients, and their idea that BITA grafting is the cause of DSWI did not reach a statistical significance level, which answered our main question that skeletonized BITA grafting could be performed in selected diabetic patients without the increased risk of DSWI. Three studies that reach statistical significance level also concluded that the use of BITA grafting is recommended in diabetic patients whose risk for DSWI is low [9,36], and BITA grafting should not be routinely denied in the absence of other short term mortality risks [42]. In our opinion, the best studies are propensity score-matched studies that categorize the patients into groups keeping all demographical data and variables in mind. These studies will enable the risk factors for DSWI to be evenly divided between the groups, and the results will be more reliable. So, the concern that either the patients in the BITA group were younger, less proportion of women, lower BMI or had fewer risk factors than the SITA group would be justified. Out of these twenty-one studies given in Table 1, nine studies also performed propensity score matching, which are given in Table 2. These matched studies showed even better results compare to unmatched in favour of BITA grafting. None of these studies reached a statistical significance level. Three studies in Table 1 Gansera et al. (2017), Momin et al. (2005), and Ran et al. (2003), also compared BITA and SITA grafting in insulin-dependent diabetes mellitus (IDDM) patients [37,44,47]. The data from Momin et al., did not support the perception that IDDM has a higher risk for DSWI in the BITA group. Gansera et al., and Ran et al., also concluded that skeletonized BITA grafting could be performed in IDDM patients without increased risk of sternal wound infection. Most of the studies of Table 1 also encouraged skeletonized BITA grafting in diabetic patients with proper patient selection, such as BITA grafts have to be avoided in obese diabetic women.

**Table 1:** Data from 21 relevant studies comparing BITA vs SITA in diabetic patients.

Serial no	Author and publishing year	Total No of patients	No of Diabetic patients	ITAs grafts in DM patients		ITAs Harvesting Technique	DSWI (%)		P-Value	References
				BITA	SITA		BITA	SITA		
1	Ji et al., 2020	2403	981	151	830	100% Skl	2	1	>0.05	[18]
2	Raza et al., 2017	1325	1315	360	965	100% Skl	1.7	3.2	0.01	[36]
3	Pevni et al., 2017	1528	1528	964	564	100% Skl	3.1	3.9	0.081	[4]
4	Gansera et al., 2017	250	250	125	125	Predominantly ped	2.4	3.2	0.722	[37]
5	Raza et al., 2014	11922	11922	938	8466	100% Skl	3.4	2.1	0.01	[9]
6	Puskas et al., 2012	3527	1445	232	1213	Mixed	1.7	1.5	0.78	[6]
7	Konstanty-kalandyk et al., 2012	147	147	38	109	100% Ped	5.2	7.3	1.00	[38]
8	Dorman et al., 2012	1107	1107	461	646	100% Skl	2.8	1.5	0.144	[39]
9	Kinoshita et al., 2010	770	423	170	170	100% Skl	2.4	1.8	0.72	[40]
10	Pusca et al., 2008	10811	3876	151	3725	Mixed	3.3	2.1	0.31	[41]
11	Savage et al., 2007	120793	120793	1732	119061	N/A	2.8	1.7	0.0004	[42]
12	Toumpoulis et al., 2006	980	980	490	490	N/A	3.3	1.2	0.050	[43]
13	Momin et al., 2005	7581	922	396	524	100% Ped	2	1.3	0.42	[44]
14	Steven et al., 2005	4382	633	214	419	100% Ped	1.4	2.2	0.5484	[7]
15	De Paulis et al., 2005	900	255	131	124	Predominantly Ped	3.5	1.6	0.4	[45]
16	Calafiore et al., 2005	558	558	200	200	Predominantly Skl	3	1.5	0.500	[46]
17	Lev-Ran et al., 2004	285	285	228	57	100% Skl	1.8	1.8	1.000	[8]
18	Lev-Ran et al., 2003	124	124	50	74	Predominantly Skl	4	2.7	1.000	[47]
19	Hirotani et al., 2003	303	303	179	124	Predominantly Ped	2.2	1.6	0.70	[48]
20	Endo et al., 2003	1131	467	190	277	100% Skl	0.5	1.1	0.65	[49]
21	Gansera et al., 2001	3671	1007	418	589	100% Ped	5	2.9	N·S	[50]

ITA: Internal thoracic arteries, BITA: Bilateral internal thoracic arteries.

SITA: Single internal thoracic arteries, DSWI: Deep sternal wound infection.

DM: Diabetes Mellitus, Skl: Skeletonized, Ped: Pedicle, N·S: Not significant.

**Table 2:** Propensity score-matched studies comparing BITA vs SITA in diabetic patients.

Serial no	Author and publishing year	Total no of Patients	BITA grafts	SITA grafts	ITAs harvesting technique	BITA DSWI (%)	SITA DSWI (%)	P-Value	References
1	Ji et al., 2020	212	105	107	100% Skl	2.9	1.9	0.337	[18]
2	Raza et al., 2017	564	282	282	100% Skl	1.4	1.4	0.7	[36]
3	Pevni et al., 2017	980	490	490	100% Skl	3.5	3.3	0.416	[4]
4	Gansera et al., 2017	250	125	125	Predominantly ped	2.4	3.2	0.722	[37]
5	Dorman et al., 2012	828	414	414	100% Skl	3.1	1.7	0.180	[39]
6	Kinoshita et al., 2010	340	170	170	100% Skl	2.4	1.8	0.72	[40]
7	Toumpoulis et al., 2006	980	490	490	N/A	3.3	1.2	0.050	[43]
8	De Paulis et al., 2005	255	131	124	Predominantly Ped	3.5	1.6	0.4	[45]
9	Calafiore et al., 2005	338	170	168	Predominantly Skl	1.2	1.8	0.500	[46]

BITA DSWI: Bilateral internal thoracic artery deep sternal wound infection.

SITA DSWI: Single internal thoracic artery deep sternal wound infection.

Skl: Skeletonized, Ped: Pedicle.

As there is no absolute indication for BITA surgery and the option is up to the surgeon's choice, specifically in diabetic patients with multi-vessel CAD. As mentioned above, the benefits of BITA compare to SITA alone or in combination with additional grafts in terms of survival, patency and, freedom from major adverse cardiac events (MACE) has been discussed by many researchers. The different configuration of ITAs during revascularization has also been studied with different results. This study aims neither to prove the superiority of BITA grafting over other groups nor to discuss the indication and contraindication of BITA grafting. Instead, it shows BITA grafting relation to the DSWI in diabetic patients.

There are many risk factors discussed in the literature for DSWI in diabetic patients, e.g. female gender, old age, obesity, DM, COPD, PVD, CKD, BITA grafting, pedicle ITAs harvesting, recent MI, urgent or emergency surgery, re-sternotomy for bleeding and redo surgeries. All the studies given in Table 1 have not thoroughly discussed the risk factors for DSWI in diabetic patients because all these studies are not purely about diabetic patients. Those who discussed different risk factors for DSWI and derived some conclusions are mentioned in Table 3. The odds ratio (OR), P-value (P), percentage, or how many folds increased risk of DSWI by any factor are also given in Table 3. Some studies did not do the statistical analysis of the risk factors for DSWI in diabetic patients and did not explain which factors are independent predictors for DSWI and which not, but though derived a result and recommend their approach. For example, Raza et al. (2017) advised that obese diabetic female need to undergo SITA + RA instead of BITA to avoid DSWI [9]. Puskas et al. (2012) recommended avoiding BITA in a morbidly obese diabetic female with pre-operative HbA1c greater than 7.5% may decrease the risk of DSWI [6]. Toumpoulis et al. (2006) supported the idea of BITA in diabetic patients whose age is 70 years or less and who do not need emergency surgery [43]. Lev-Ran et al. (2003) suggested that BITA should be avoided in obese, IDDM patients with BMI > 29 kg/m^2^ associated with COPD or undergoing emergency surgery. They think obese diabetics have a 7.7-fold increase risk of DSWI [47].

**Table 3:**
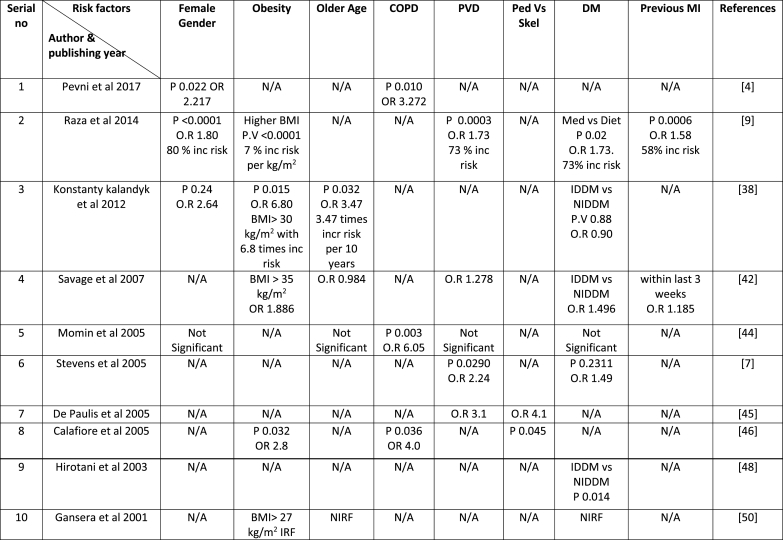
Risk factors for DSWI in diabetic patients with its P-Value (P) and Odds Ratio (OR).

COPD: Chronic obstructive pulmonary disease; PVD: Peripheral vascular disease; DM: Diabetes mellites; N/A: Not available.

IDDM: Insulin-dependent diabetes mellitus; NIDDM: Non-insulin-dependent diabetes mellitus; BMI: Body mass index.

IRF: Independent risk factor; NIRF: Not independent risk factor.

From our analysis of all these studies mentioned in Table 1 and its risk factors tabulated in Table 3, the most prominent risk factors for DSWI in diabetic patients undergoing BITA revascularization are obesity, COPD, and female gender.

Most surgeons avoid BITA grafting because the patient only has diabetes without many more risk factors for DSWI. Diabetes mellitus may be a risk factor for DSWI, and outcomes of patients having diabetes may not be as good as those who do not have diabetes mellitus. Still, BITA grafting having almost similar risk for DSWI in diabetic patients as SITA grafting. From our current study, we concluded that if the patient does not have a high-risk profile for DSWI, such as obese diabetic female with COPD, or the combination of few risk factors discussed above, the skeletonized BITA grafting can be performed safely in diabetic patients with multi-vessel CAD. As there is not much data available on the topic and there are controversies in the studies available, further research is warranted especially randomized control trials (RCT), to know the exact risk of DSWI in diabetic patients undergoing CABG using ITAs.

## 5 Conclusion

BITA grafts have better patency rate, and long-term outcomes compare to SITA graft alone or in combination with other grafts in general, diabetic as well as in high-risk population. Deep sternal wound infection is a multifactorial process, and to point out a single culprit and make decisions based on it may not be justice to patients. Factors such as obesity, COPD, female gender, old age, diabetes mellitus, renal failure and, peripheral vascular disease may play a role in its development. We found obesity, COPD and female gender the most prominent risk factors for DSWI in diabetic patients. The surgeon who intends to perform single internal thoracic artery graft in combination with other grafts in a diabetic patient for the sake of DSWI avoidance would better perform skeletonized bilateral internal thoracic artery grafts, as its risk of deep sternal wound infection is almost similar. This decision is valid if diabetes mellitus is the only risk factor for DSWI. Still, if other risk factors such as obesity, COPD and female gender are also present, a much wise decision has to be made.

## Ethical approval

Ethical approval is not applicable for Review article.

## Sources of funding

This study was not financially supported by any organization.

## Author contribution

Conception and design: M M and Y Z: Provision of study materials: M M and Y Z; Collection and assembly of data: M M and X F; Data analysis and interpretation: All authors; Manuscript writing: All authors; Final approval of manuscript: All authors.

## Trial registry number

1. Name of the registry: Not applicable.

2. Unique Identifying number or registration ID: not applicable.

3. Hyperlink to your specific registration (must be publicly accessible and will be checked):

## Guarantor

Dr.Yuan Zhao.

## Consent for publication

Not applicable

## Declaration of competing interest

All authors have no conflicts of interest to declare.

## Abbreviations

BITA: Bilateral internal thoracic arteries
SITA: Single internal thoracic artery
ITAs: Internal thoracic arteries
DSWI: Deep sternal wound infection
CABG: Coronary artery bypass grafting
CAD: Coronary artery disease
COPD: Chronic obstructive pulmonary disease
PVD: Peripheral vascular disease
CRF: Chronic renal failure
